# Supplementary material for: Structural basis for Rad54- and Hed1-mediated regulation of Rad51 during the transition from mitotic to meiotic recombination
Source: bioRxiv. 2025 Mar 26:2025.03.26.645561. Preprint. [Version 1] doi: 10.1101/2025.03.26.645561 (PMC11974805; doi:10.1101/2025.03.26.645561)
Supplement: Supplement 2 [file NIHPP2025.03.26.645561v1-supplement-2.pdf]

bioRxiv preprint doi: https://doi.org/10.1101/2025.03.26.645561; this version posted March 26, 2025. The copyright holder for this preprint (which was not certified by peer review) is the author/funder, who has granted bioRxiv a license to display the preprint in perpetuity. It is made available under aCC-BY 4.0 International license.

# Figure S1

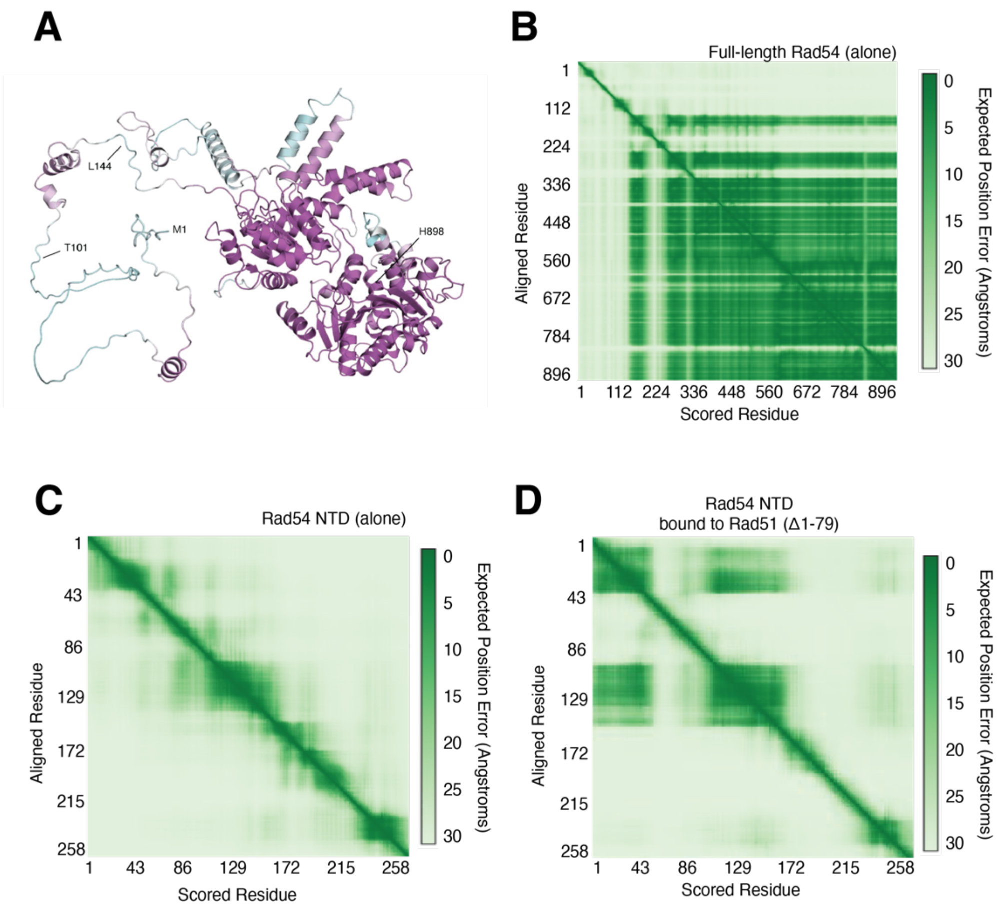

bioRxiv preprint doi: https://doi.org/10.1101/2025.03.26.645561; this version posted March 26, 2025. The copyright holder for this preprint (which was not certified by peer review) is the author/funder, who has granted bioRxiv a license to display the preprint in perpetuity. It is made available under aCC-BY 4.0 International license.

**Figure S2**

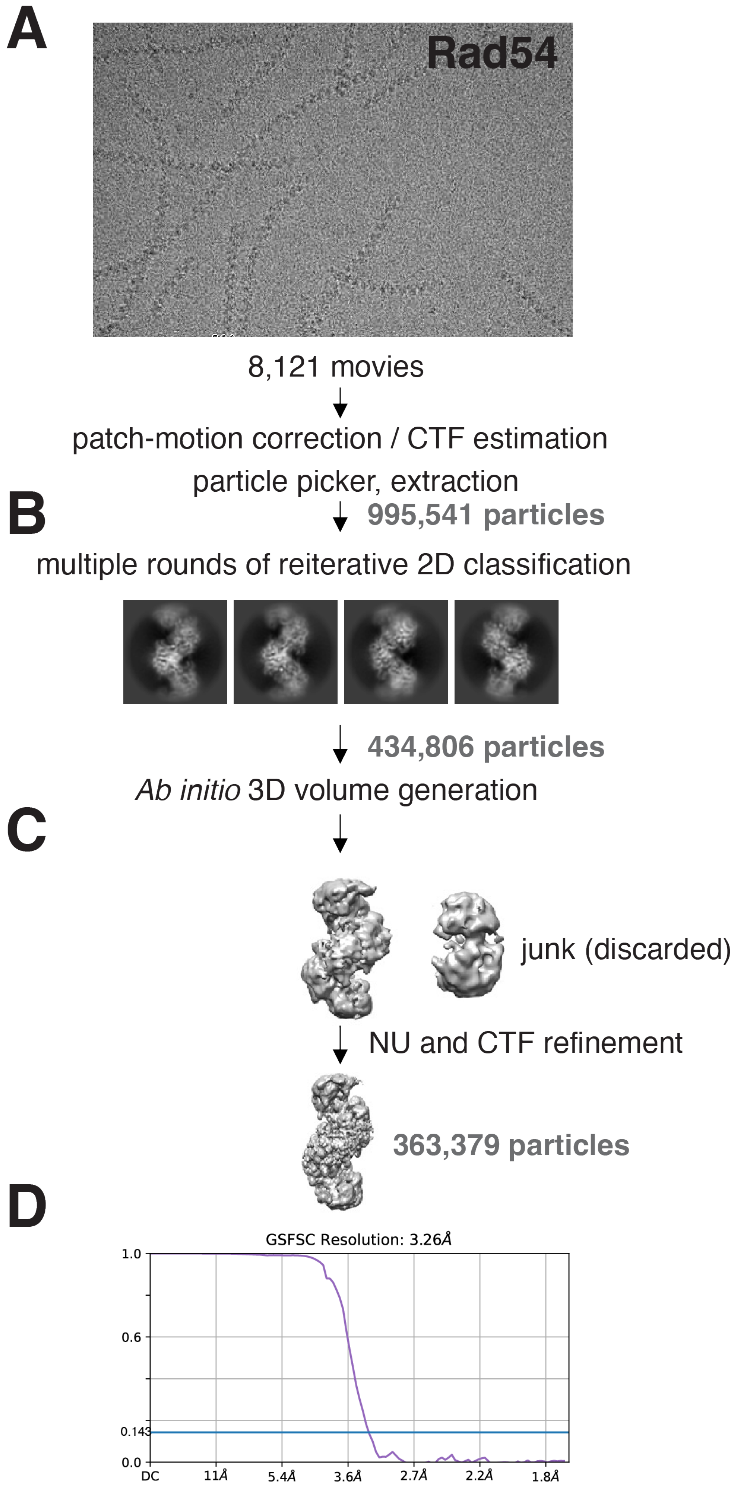

bioRxiv preprint doi: https://doi.org/10.1101/2025.03.26.645561; this version posted March 26, 2025. The copyright holder for this preprint (which was not certified by peer review) is the author/funder, who has granted bioRxiv a license to display the preprint in perpetuity. It is made available under aCC-BY 4.0 International license.

# Figure S3

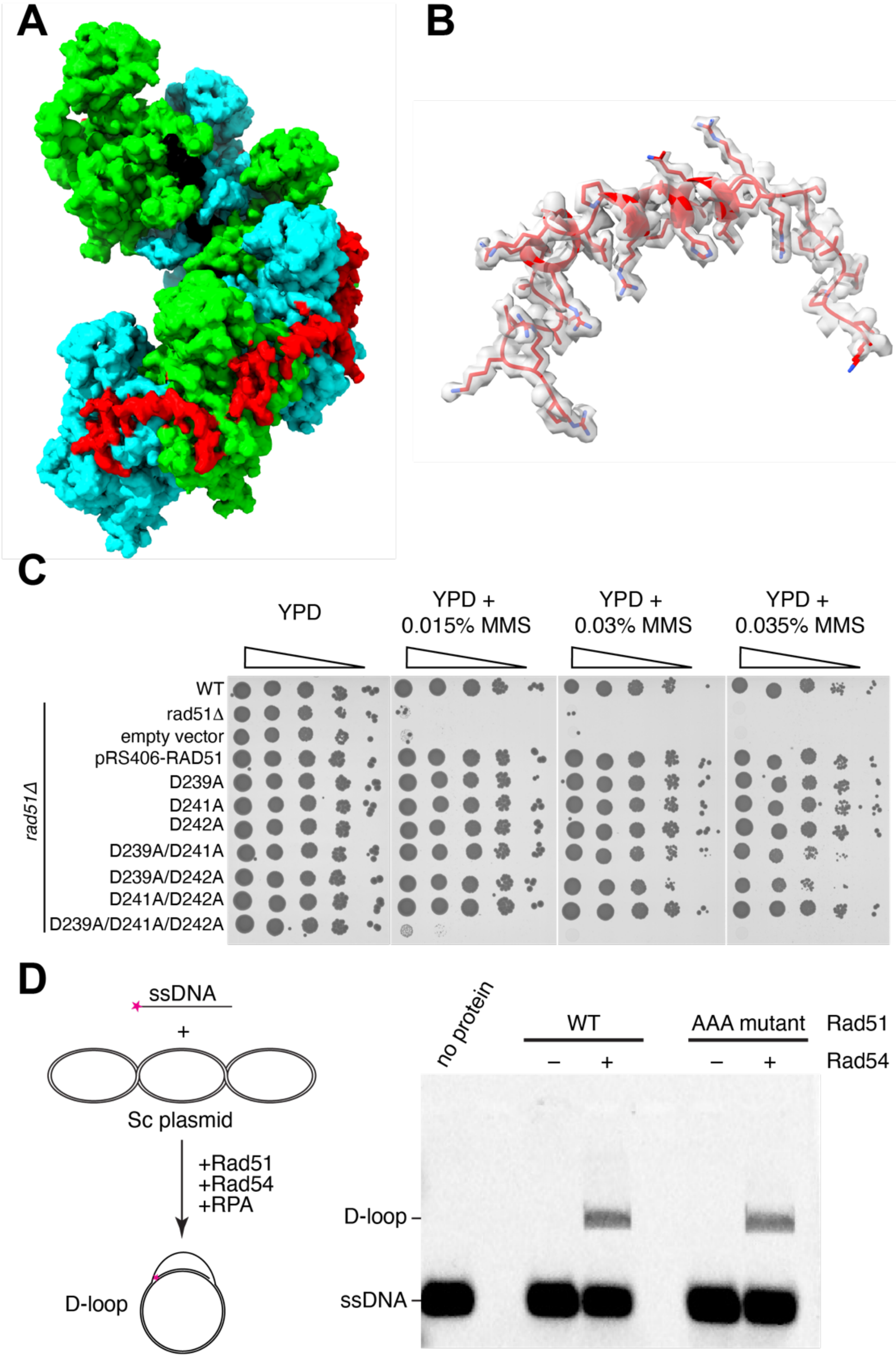

bioRxiv preprint doi: https://doi.org/10.1101/2025.03.26.645561; this version posted March 26, 2025. The copyright holder for this preprint (which was not certified by peer review) is the author/funder, who has granted bioRxiv a license to display the preprint in perpetuity. It is made available under aCC-BY 4.0 International license.

# Figure S4

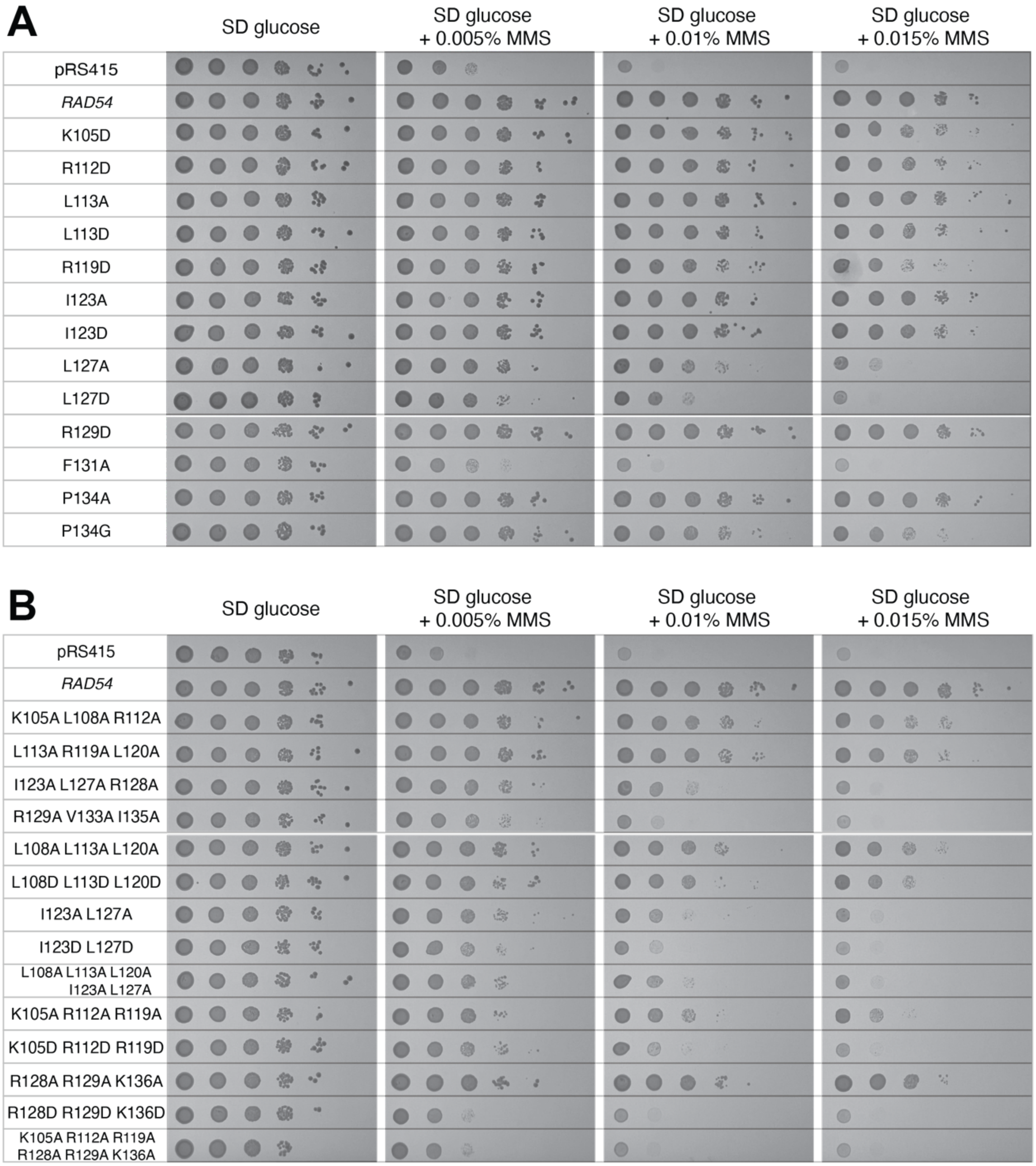

bioRxiv preprint doi: https://doi.org/10.1101/2025.03.26.645561; this version posted March 26, 2025. The copyright holder for this preprint (which was not certified by peer review) is the author/funder, who has granted bioRxiv a license to display the preprint in perpetuity. It is made available under aCC-BY 4.0 International license.

**Figure S5**

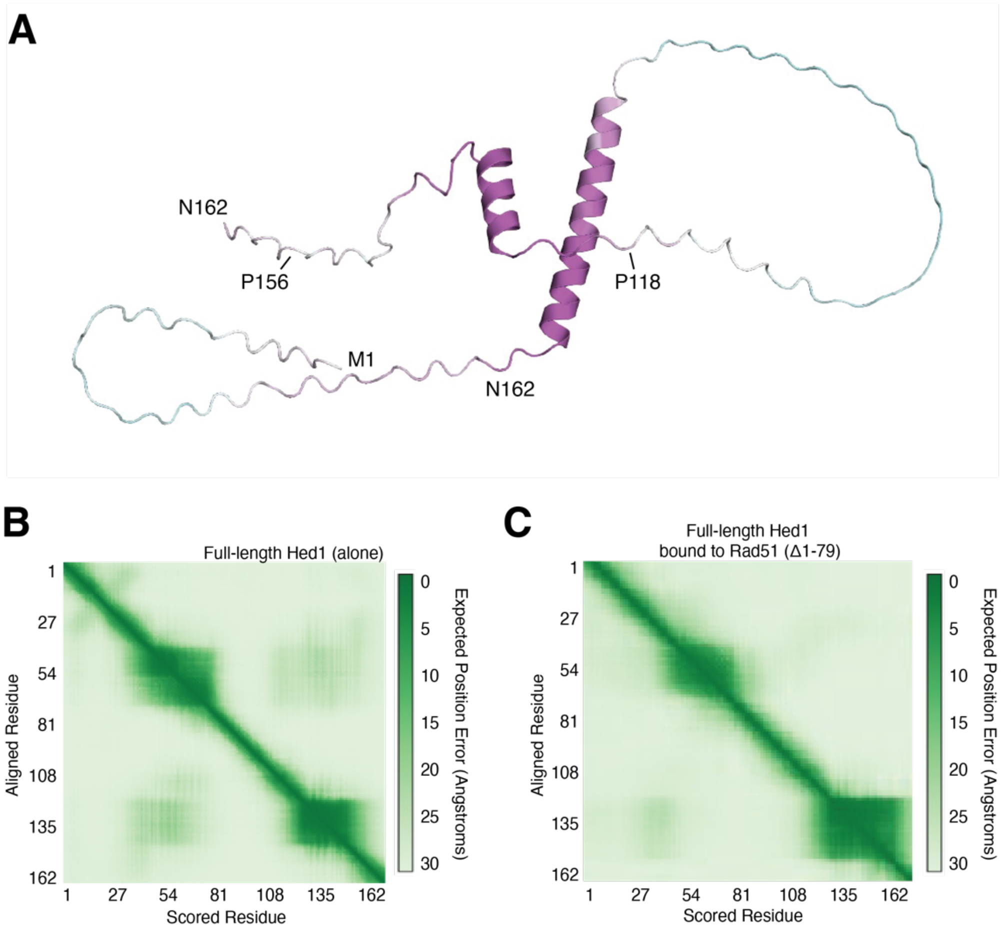

bioRxiv preprint doi: https://doi.org/10.1101/2025.03.26.645561; this version posted March 26, 2025. The copyright holder for this preprint (which was not certified by peer review) is the author/funder, who has granted bioRxiv a license to display the preprint in perpetuity. It is made available under aCC-BY 4.0 International license.

**Figure S6**

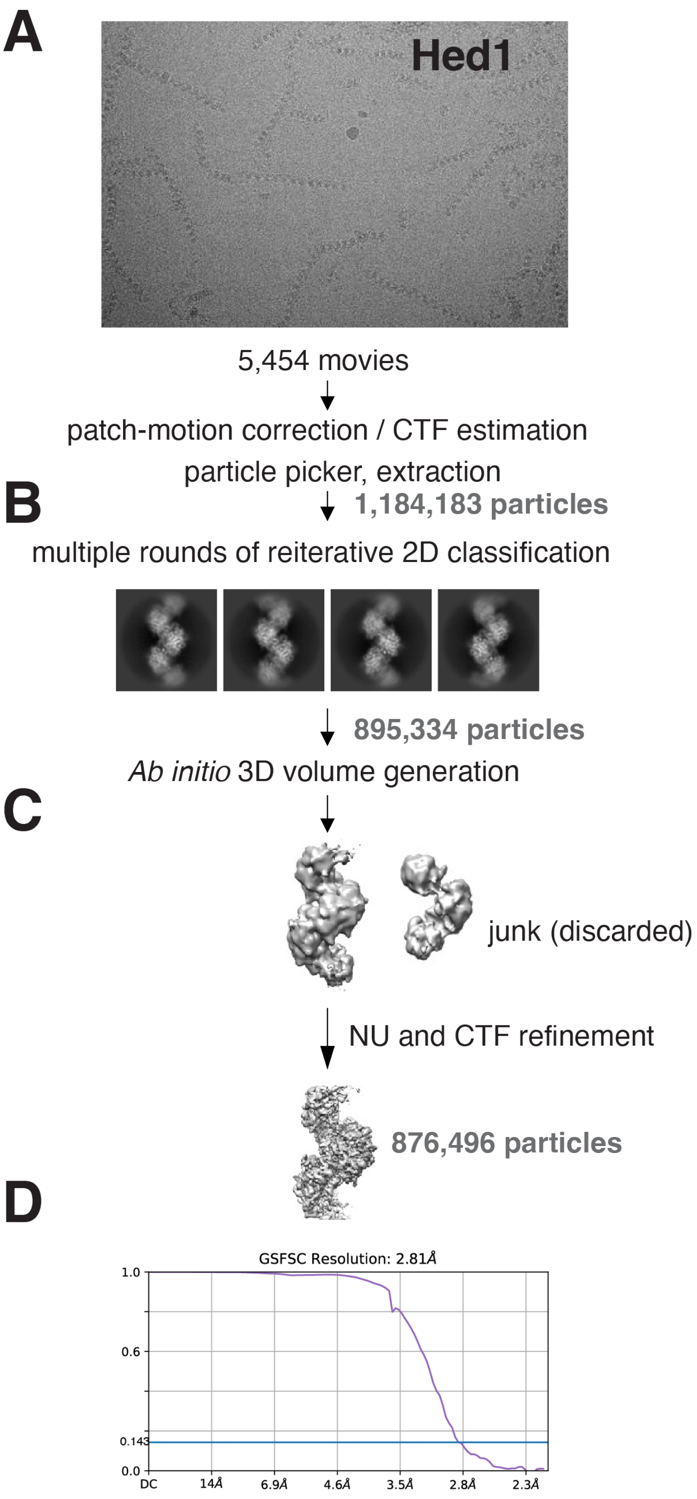

bioRxiv preprint doi: https://doi.org/10.1101/2025.03.26.645561; this version posted March 26, 2025. The copyright holder for this preprint (which was not certified by peer review) is the author/funder, who has granted bioRxiv a license to display the preprint in perpetuity. It is made available under aCC-BY 4.0 International license.

**Figure S7**

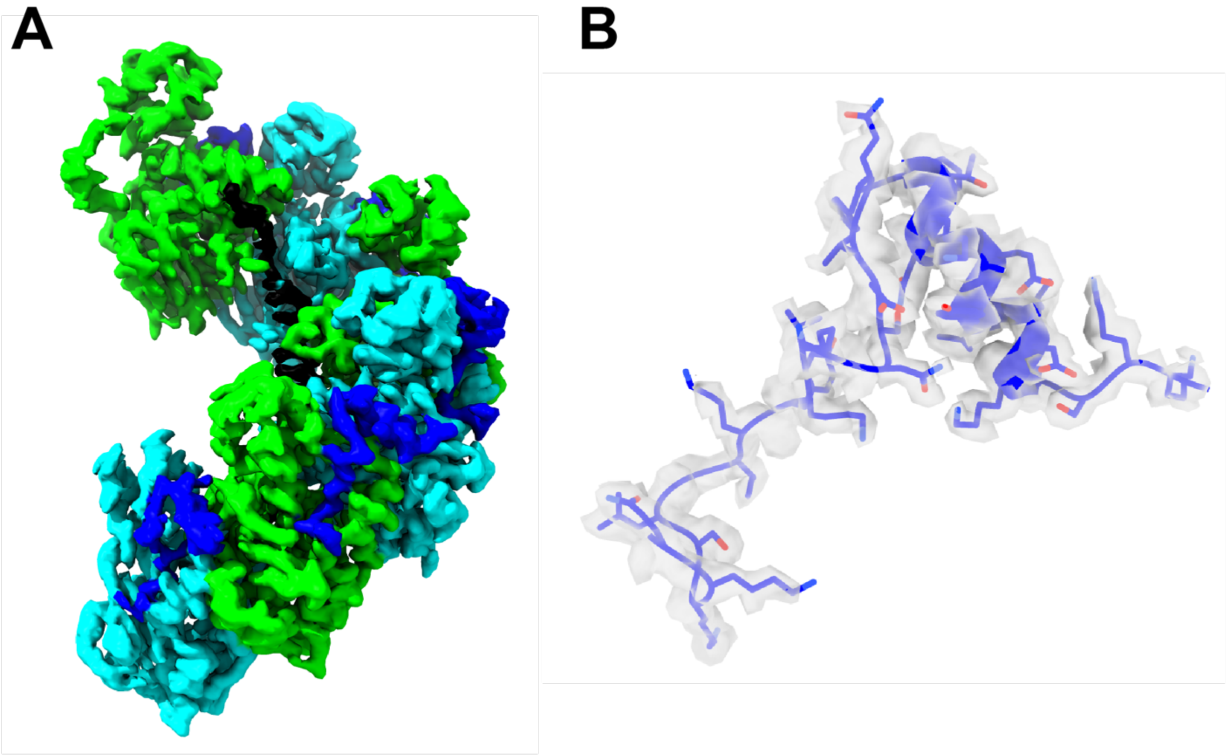

bioRxiv preprint doi: https://doi.org/10.1101/2025.03.26.645561; this version posted March 26, 2025. The copyright holder for this preprint (which was not certified by peer review) is the author/funder, who has granted bioRxiv a license to display the preprint in perpetuity. It is made available under aCC-BY 4.0 International license.

# Figure S8

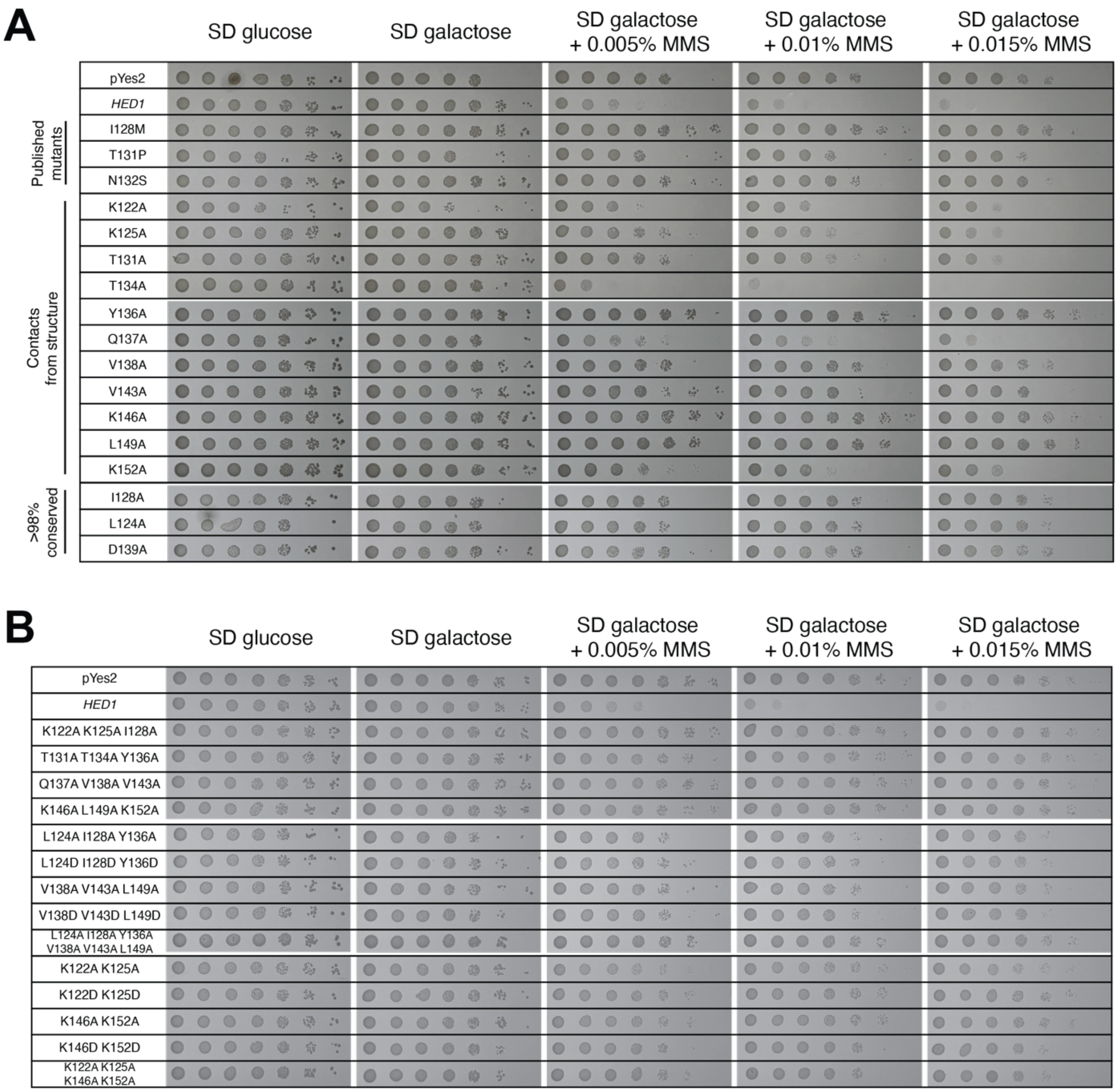

bioRxiv preprint doi: https://doi.org/10.1101/2025.03.26.645561; this version posted March 26, 2025. The copyright holder for this preprint (which was not certified by peer review) is the author/funder, who has granted bioRxiv a license to display the preprint in perpetuity. It is made available under aCC-BY 4.0 International license.

**Figure S9**

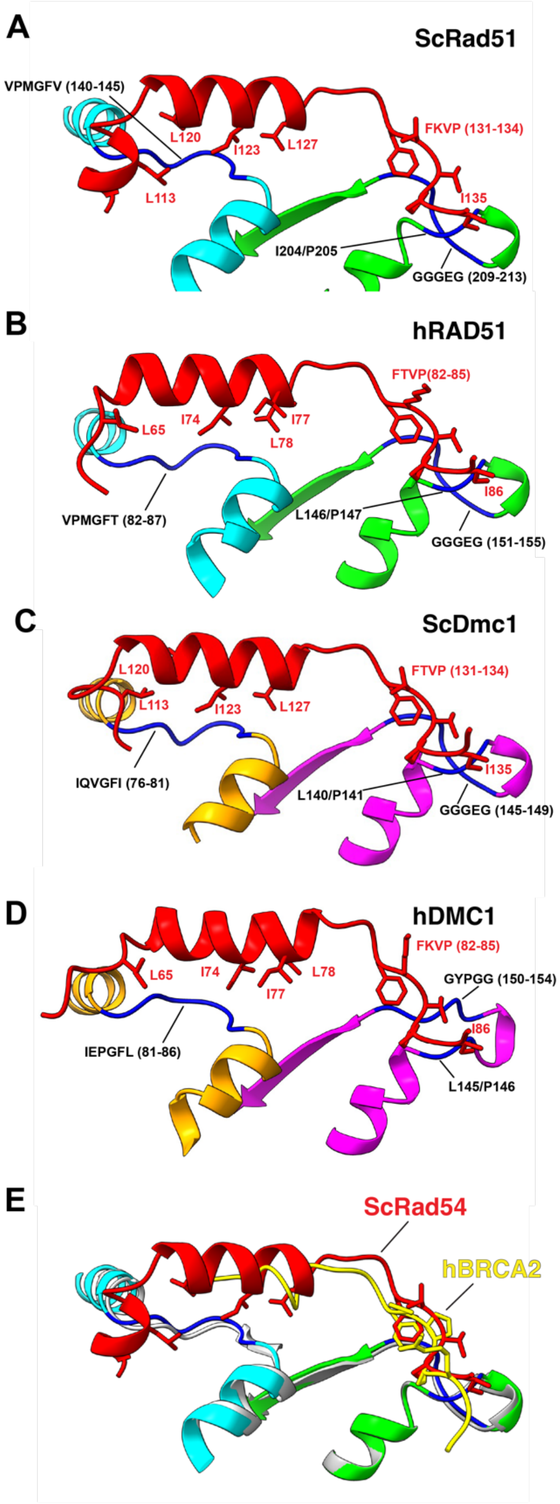

bioRxiv preprint doi: https://doi.org/10.1101/2025.03.26.645561; this version posted March 26, 2025. The copyright holder for this preprint (which was not certified by peer review) is the author/funder, who has granted bioRxiv a license to display the preprint in perpetuity. It is made available under aCC-BY 4.0 International license.

## Table S1.

| | Rad51_Rad54 PDB: 9E6L (EMD-47572) | Rad51_Hed1 PDB: 9E6N (EMD-47573) |
|---|---|---|
| **Data collection and processing** | | |
| Microscope | Titan Krios | Titan Krios |
| Voltage (keV) | 300 | 300 |
| Detector | K3 | K3 |
| Magnification | 105,000 | 85,000 |
| Voltage (kV) | 300 | 300 |
| Electron exposure ($e^-$/Å$^2$) | 59.51 | 51.21 |
| Defocus range (μm) | -0.8 to -2.5 | -0.8 to -2.5 |
| Pixel size (Å) | 0.844 | 1.083 |
| | | |
| Initial particles picked | 995,541 | 1,184,183 |
| Final particles used | 363,379 | 876,496 |
| Map resolution (Å)<br>FSC threshold | 3.3<br>0.143 | 2.8<br>0.143 |
| Map resolution range (Å) | 3.3-3.7 | 2.8-3.3 |
| | | |
| **Refinement** | | |
| Model resolution (Å)<br>FSC threshold | 3.3<br>0.143 | 2.8<br>0.143 |
| *Model composition*<br>Non-hydrogen atoms<br>Protein residues<br>Ligands | <br>16,562<br>2,092<br>ATP, MG | <br>16,759<br>2,115<br>ATP, MG |
| *R.m.s. deviations*<br>Bond lengths (Å)<br>Bond angles (°) | <br>0.002<br>0.453 | <br>0.003<br>0.467 |
| *Validation*<br>MolProbity score<br>Clash score<br>Rotamer outliers (%) | <br>1.27<br>5.14<br>0 | <br>1.25<br>4.81<br>0 |
| *Ramachandran plot*<br>Favored (%)<br>Allowed (%)<br>Outliers (%) | <br>98.69<br>1.26<br>0 | <br>98.42<br>1.58<br>0 |
